# Syntaxin 16 Regulates Lumen Formation during Epithelial Morphogenesis

**DOI:** 10.1371/journal.pone.0061857

**Published:** 2013-04-23

**Authors:** Jae-Joon Jung, Shivangi M. Inamdar, Ajit Tiwari, Ding Ye, Fang Lin, Amit Choudhury

**Affiliations:** 1 Department of Anatomy and Cell Biology, University of Iowa, Iowa City, Iowa, United States of America; 2 Orthopaedics and Rehabilitation, University of Iowa, Iowa City, Iowa, United States of America; Emory University School of Medicine, United States of America

## Abstract

The formation and maintenance of cell-cell junctions, both under physiological and pathological conditions, requires the targeting and trafficking of junctional proteins. Proteins of the syntaxin (Stx)-family localize to a variety of subcellular membranes and contribute to intracellular transport of cargo by regulating vesicle fusion events at these sites. Unlike plasma membrane localized Stxs, the roles of endosome- and Golgi-localized stx proteins in epithelial morphogenesis are less understood. Here we show that Stx16– an endosome- and Golgi-localized target-membrane soluble N-ethylmaleimide attachment protein receptor (t-SNARE) that plays a role in membrane trafficking between these compartments – is essential for lumen development. In cultured Madin Darby Canine Kidney (MDCK) cells, Stx16 was selectively upregulated as sparsely plated cells attained confluency. Stx16-depleted confluent monolayers consistently showed lower transepithelial resistance than control monolayers, and failed to maintain endogenous and ectopically expressed E-cadherin at the adherens junctions due to decreased recycling. We further found that whereas cysts formed by MDCK cells cultured in Matrigel have a single hollow lumen, those formed by stx16-depleted counterparts had multiple lumens, due to abnormal orientiation of the mitotic spindle. Finally, a similar role for stx16 function *in vivo* is indicated by our analysis of pronephric-duct development in zebrafish expressing the *claudinB:lynGFP* transgene; lack of stx16 function in this structure (in *stx16-*morphant embryos) led to the development of enlarged, torturous pronephric ducts with more than one lumen. Taken together, our *in vitro* and *in vivo* studies establish a role for Stx16 in maintaining the integrity of cell-cell junctions, and thereby in morphogenesis of the kidney epithelial lumen.

## Introduction

The formation of polarized epithelia requires a functional apical junctional complex, major components of which are adherens junctions (AJs) and tight junctions (TJs). In epithelia, the AJs promote cell-cell adhesion and coordinate the changes in cell shape that are necessary for morphogenesis and organogenesis. An AJ component that is key to these functions is E-cadherin, a calcium-dependent, homophilic, cell-to-cell adhesion receptor located in the basolateral domain. AJ-localized E-cadherin is linked to the actin cytoskeleton by scaffolding proteins such as the catenins. Given that it contributes to AJ formation as well as to the maintenance of epithelial integrity during tissue homeostasis and remodeling, its activities must be precisely regulated. E-cadherin regulation is achieved in part by the transport of cadherin- and catenin-containing vesicles to and from the plasma membrane (PM) via precisely tuned exocytic and endocytic events [1].

Lumen formation was a crucial step in metazoan evolution, as it enables essential functions such as nutrient uptake, gas exchange, and circulation. In addition, it is a key step in organogenesis, specifically with respect to establishing the organ’s architecture and function [2]. In spite of a high degree of morphogenetic diversity among metazoan species, the end result of lumen formation is always a structure in which the apical surface of the epithelial cell faces the lumen [3]. Establishment and expansion of the apical lumen is a key step during tissue morphogenesis [3], [4]. MDCK cells are a classic mammalian system for analyzing the assembly of E-cadherin-based AJs, as well as the function of E-cadherin in epithelial polarization [5], [6], [7], [8]. When grown in three-dimentional (3D) culture in an extracellular matrix (collagen I or Matrigel), MDCK cells proliferate and organize into cysts, hollow, spherical structures in which a monolayer of highly polarized epithelial cells surround a single, central lumen [2], [4]. Although tissue-culture models have provided important insights into the molecular mechanisms underlying lumen formation, how these mechanisms relate to epithelial development within the kidney remains to be established. Thus development of the zebrafish pronephros has been developed as simplified model system for carrying out *in vivo* studies of kidney morphogenesis to complement the tissue-culture studies [9].

To date, the role of vesicle trafficking in the control of membrane remodelling during cell and tissue morphogenesis has received little attention. In eukaryotic cells, most membrane-fusion steps require soluble *N*-ethylmaleimide-sensitive factor attachment protein receptors (SNAREs) [10], [11]. The SNAREs are classified into two major classes based on the presence of a glutamine (Q SNAREs or t-SNAREs) or an arginine (R SNAREs or v-SNAREs) in the center of the SNARE motif. Unlike plasma membrane localized Stxs (Stx3, Stx4), the roles of other Stx proteins, e.g., of those localized at the endosomes and Golgi, in formation of the epithelial lumen has not been explored. Stx16 is a member of the t-SNARE protein family, localizes primarily to the Golgi apparatus, and is involved in the transport of cargo molecules from endosomes to the Golgi apparatus [12], [13], [14], [15], [16], [17]. However, the role of this SNARE protein in epithelial morphogenesis remains poorly understood. We set out to do so given that our analysis of MDCK cells shows that level of Stx16 increases as cells attain confluency and that it plays a role in maintaining E-cadherin at cell-cell junctions and the paracellular ion barrier. Moreover, we show that during 3D cystogenesis, Stx16-depleted cells show abnormal spindle orientation and develop multiple lumens, suggesting that this Stx plays a role in establishing a single apical lumen. As proof of concept that Stx16 is indeed required for kidney morphogenesis *in vivo*, we show that zebrafish *stx16*-morphants show aberrant pronephric duct morphogenesis.

## Methods

### Reagents

Monoclonal antibodies (MAbs) against EEA1, Stx6, and TGN46, and growth factor-reduced Matrigel were obtained from BD Biosciences (San Jose, CA). Rabbit polyclonal Abs (PAbs) against Stx6 and Stx16, and the mouse MAb against Stx16 were purchased from Synaptic Systems (Goettingen, Germany). Fugene6 HD transfection reagent, protease inhibitor and phosphatase-inhibitor cocktail tablets were obtained from Roche Diagnostics (Indianapolis, IN). Alexa Fluor-conjugated secondary Abs were obtained from Invitrogen, Molecular Probes (Carlsbad, CA). Vectashield mounting medium was purchased from Vector laboratories, Inc. (Burlingame, CA). The xxx α-tubulin MAb and the rabbit pan-cadherin PAb were purchased from Sigma Chemical Co. (St. Louis, MO). Rabbit polyclonal anti-PKCζ antibody was obtained from Santa Cruz Biotechnology (Santa Cruz, CA). Fluorescently-labeled Phalloidin was obtained from Life Technologies (Carlsbad, CA). Protein A&G sepharose beads and Amplify-fluorographic Reagent were purchased from GE Healthcare. SuperSignal West Femto ECL reagent was obtained from Thermo Scientific, USA. Plasmid construct for human E-cadherin-GFP was obtained from Dr. Jennifer L. Stow (University of Queensland, Australia) and has been described before [18].

### Cell Culture

MDCK II (MDCK) cells were maintained in low-glucose DMEM (LG-DMEM) with 1.8 mM Ca^2+^, 1 g/l sodium bicarbonate, 10% fetal bovine serum (FBS; Cell Generation, Fort Collins, CO), penicillin, streptomycin, and gentamicin, and were grown at 37°C with 5% CO_2_. sh*STX*16 cells were generated by stable integration of shRNAs targeting canine STX16 (STX16: sense, 5′-TCGGAAGATGCTTGTGATTTT-3′), respectively. shRNAs were delivered via transduction with recombinant lentiviral vectors generated using the 2nd generation packaging system (pCMV-dR8.2, pCMV-VSVG) method. Negative controls were generated by transducing MDCK cells with lentiviral vectors encoding a scrambled shRNA (sense, 5′-CCTAAGGTTAAGTCGCCCTCGC-3′; scr-shRNA) and selecting them in puromycin.

### MDCK Cyst-formation Assay

Five µl of growth factor-reduced Matrigel were added to each well of an 8-well glass chamber slide and left to solidify at 37°C for at least 15 min prior to cell preparation. Five-thousand MDCK cells were prepared to a single cell suspension in 400 µl of complete medium, DMEM supplemented with 10% FBS and 1% penicillin-streptomycin (PS), mixed with 2% Matrigel and then placed in each well. The cells were incubated for the indicated time periods in complete medium containing 2% Matrigel, which was changed every other day.

### Measurement of Transepithelial Resistance

MDCK cells were plated onto 0.4 µm pore and 12 mm Transwell polycarbonate filters at 5.5×10^5^ density per filter in complete medium (DMEM supplemented with 10% FBS and 1% PS). The formation of TJs was evaluated as the transepithelial electrical resistance (TER) across the cell monolayer [19], [20], with high electrical resistance representing well-developed TJs, and a decrease in TER disruption of junction integrity [19]. TER was first measured at 12 h after plating and continued until the values plateaued, using the Millicell electrical resistance system (Millipore). All measurements were performed in triplicate and the final values were obtained by the subtraction of values for a blank and multiplication by the surface area of the filter (ohms•cm^2^). For the Ca^2+^ switching experiments, the complete medium (high-Ca^2+^) was replaced with HBSS containing 2 mM ethylene glycol tetra acetic acid (EGTA) and 5% dialyzed FBS for 30 min in a cell culture incubator, followed by replacement with complete medium. TER was measured at the indicated time points. For each filter, three independent readings were taken per time point, using a Millicell (Millipore) electrical resistance device. Final values were obtained by subtracting the averaged blank value from the averaged sample reading and multiplying by the surface area of the filter. The final results are expressed as ohms/cm^2^. The intrinsic resistance of the polycarbonate filters determined by measuring TER in the absence of a cell monolayer.

### Immunofluorescence Assay and Image Analysis

Microscopy studies were performed as described previously [21]. Briefly, cells grown on acid-washed glass coverslips were fixed with 4% paraformaldehyde in Dulbecco’s modified phosphate-buffered saline (PBS) for 25 min at room temperature (RT), after which the reaction was quenched by adding 100 mM glycine in PBS and incubating for a further 15 min at RT. The cells were then permeabilized with 0.1% Triton X-100 in PBS for 2 min at RT, blocked with PBS containing 5% glycine and 5% normal goat or donkey serum for 60 min, and incubated with primary Abs overnight at 4°C. Coverslips were incubated for 1 h in a 1∶200 dilution of either Alexa Fluor 488- or Alexa Fluor 594-conjugated secondary A, and mounted using Vectashield mounting medium containing DAPI. Fluorescence images were acquired using a Zeiss LSM 700 inverted confocal microscope (Carl Zeiss) with a Plan-Apochromat 63×/1.40 oil objective or Plan-Apochromat 40×/1.3 oil objective (Zeiss) in the x-, y- and z-planes. Epifluorescence images were acquired using a Leica spinning-disk confocal microscope equipped with a Hamamatsu EM-CCD digital camera (Hamamatsu Photonics). The acquired images were processed using “Metamorph” image processing software (Molecular Device Corp., Downingtown, PA). For a given experiment all photomicrographs for each fluorophore were exposed and processed identically. Background fluorescence correction was done using unlabeled specimens. For double and triple Ab-labeling experiments, control samples were labeled identically with the individual fluorophores and exposed identically to the dual/triple-labeled samples at each wavelength to verify that there was no crossover between emission signals for different channels at the exposure settings used. For immunofluorescence analysis of zebrafish embryos, transverse frozen sections were prepared, fixed in 4% PFA overnight at 4°C, and washed three times with PBS. The embryos were incubated in a series of sucrose solutions of concentrations increasing from 5% to 20%, and then embedded and frozen in OCT solution, sectioned using a cryostat, and subjected to antibody labeling.

Zebrafish microscopy-ISH Images were taken using a Leica M165FC Stereomicroscope with a Leica DFC290 Color Digital Camera or Nikon Microphot-FX microscope. Live embryos were mounted in 1% methylcellulose and photographed using a Leica DMI 6000 microscope with a 5×/NA 0.15 objective. Confocal images were acquired using a laser-scanning confocal inverted microscope (LSM700, Carl Zeiss, Inc.) with 40×/NA 1.3 oil objective.

### Cyst Immunofluorescence

Immunofluorescence (IF) of MDCK cysts was performed according to a previously published protocol (Debnath et al., Methods, 2003). In brief, the cysts were fixed with 2% paraformaldehyde in PBS for 20 min at room temperature (RT), permeabilized with 0.5% Triton X-100 in PBS for 10 min at 4°C, and rinsed three times for 10 min each with PBS/glycine (130 mM NaCl, 7 mM Na_2_HPO_4_, 3.5 mM NaH_2_PO_4_, and 100 mM glycine). For primary blocking, the cysts were incubated in IF buffer (130 mM NaCl, 7 mM Na2HPO4, 3.5 mM NaH2PO4, 7.7 mM NaN3, 0.1% BSA, 0.2% Triton X-100, and 0.05% Tween-20) with 10% normal goat serum (NGS) for 1.5 h, followed by secondary blocking in IF buffer with 10% NGS and 20 µg/ml goat anti-mouse F(ab’)2 fragments for 40 min. The cysts were then incubated with primary antibody in secondary blocking solution overnight at 4°C. After washing three times with IF buffer for 20 min each at RT, the cysts were incubated with secondary antibody in primary blocking solution for 50 min at RT. After three additional washes with IF buffer, the cysts were mounted with VectaShield that contains DAPI for nuclear staining. All images were photographed on a Zeiss 510 LSM confocal microscope. To quantify cysts with abnormal lumen formation, 100–150 cysts were examined under each condition.

### Immunoblotting

Cultured cells were washed twice with ice-cold PBS and lysed in RIPA buffer supplemented with inhibitors of proteases and phosphatases for 30 min on ice. They were then centrifuged at 13000g for 20 min before supernatants were collected. The protein concentration was estimated using the Bio-Rad DC Protein Assay kit (Bio-Rad Laboratories). 10–25 µg of protein per lane were resolved by SDS-PAGE and transferred to a PVDF membrane. After blocking with 5% (w/v) non-fat dried skimmed milk powder in TBST buffer, the membrane was incubated with the appropriate primary Abs at 4°C overnight, and then with secondary Ab for 1h at room temperature. Ab binding was visualized by developing the blot with enhanced chemiluminescence reagent. The bands were visualized using ChemiDocIt (UVP), and then analyzed using ImageJ 1.42q software (http://rsbweb.nih.gov/ij/). For preparing zebrafish embryo lysates, equal number of embryos were manually deyolked and homogenized in 2× SDS sample buffer, resolved by SDS-PAGE, and immunoblotted.

### E-cadherin Recycling Assay

MDCK cells were plated onto 0.4 µm pore and 24 mm Transwell polycarbonate filters at 5×10^6^ density per filter in complete medium (DMEM supplemented with 10% FBS and 1% PS). After 12 h of cell seeding, MDCK cells on filters were incubated with HBSS containing 2 mM EGTA for 5 min and then washed with PBS. Apical and basal chambers were incubated with membrane-impermeant biotinylation reagent (NHS-SS-biotin; Pierce) at 4°C as described previously [22]. Free biotin was then quenched using 50 mM NH_4_Cl (in PBS) for 15 min (4°C). Samples were then incubated at 16°C for an additional 30 min in Ca^+2^ free PBS, to allow internalized E-cadherin to accumulate in endosomes. Biotin remaining at the cell surface was removed by reduction with sodium 2-mercaptoethanesulfonate (MesNa). MesNa was quenched by the addition of 20 mM iodoacetamide for 10 min. The cells were washed twice in ice-cold PBS. Intracellular, biotin-labeled E-cadherin were further chased at 37°C in Ca^+2^ free PBS, to allow E-cadherin to recycle to the PM. The extent of E-cadherin recycling was assessed after each time point of the 37°C chase; in this case, cells were immediately returned to ice at the time point, and biotin was removed from the recycling pool by reduction with MesNa. The levels of biotinylated E-cadherin were determined using a capture ELISA assay using cell lysates prepared in RIPA buffer as described previously [23].

### Capture ELISA

Maxisorb 96-well plates (Invitrogen) were coated overnight with 5 µg/ml of the E-cadherin Ab in 0.05 M Na_2_CO_3_ (pH 9.6) at 4°C, and blocked in PBS containing 0.05% Tween 20 (PBS-T) with 5% BSA for 1 h at RT. E-cadherin was captured by overnight incubation of 50 µl of cell lysate at 4°C. Unbound material was removed by extensive washing with PBS-T, after which streptavidin-conjugated horseradish peroxidase (Amersham Biosciences) in PBS-T containing 1% BSA was placed into each well; samples were then incubated for 1 h at 4°C. Following subsequent washing, biotinylated integrins were detected using a chromogenic reaction with ortho-phenylenediamine.

### Zebrafish Maintenance

Wild-type (AB*/Tuebingen) and transgenic *Tg(cldnb:lynEGFP)* [24], [25] strains were maintained under standard conditions at the University of Iowa animal facility. Embryos were obtained by natural mating and staged according to morphology, or hours post fertilization (hpf) at 28.5, as described previously [26].

### Ethics Statement

All zebrafish related studies were reviewed and approved by University of Iowa Animal Care and Use Committee (IACUC).

### Whole-mount *in situ* Hybridization

Full-length zebrafish *stx16* was cloned into the pCR-Blunt II-TOPO vector using the Zero Blunt TOPO PCR cloning kit (Life Technologies). The plasmid DNA was linearized with Hind III or Not I to generate sense and antisense RNA probes, respectively. Digoxigenin-labeled RNA probes were synthesized by *in vitro* transcription, and whole-mount *in situ* hybridization (ISH) was performed as described [27], [28]. After ISH, the embryos were re-fixed in 4% PFA and sectioned to 10 µm thickness, as described previously [29].

### Morpholino Injections

Morpholino antisense oligonucleotides (MO) targeting zebrafish *syntaxin 16* (*stx16*) were designed and synthesized by Gene Tools, LLC (Philomath, OR). Two separate MOs targeting the AUG region (5′-CATCGGTCAGTCGCCGAGTTGCCAT-3′, +1 to +25) and the splice acceptor site of intron 3-exon 4 (5′-ACTGAATCTGAGGGAACAAGGAATT-3′) were used. The effects of the AUG-targeting MO (MO^AUG^) and the mRNA splicing-targeting MO (MO^sp^) were confirmed by immunoblotting and RT-PCR, respectively. The *stx16*-MOs were co-injected with the *p53*-MO into embryos at the one- to two-cell stages, to reduce toxicity as reported previously [30]. RNAs were prepared from control uninjected and *stx16*-MO^sp^ injected embryos using TRIzol reagent (Life Technologies) and RT-PCR was performed using a primer set that bind to exon 3 (5′-TTGGATCCAGAGGCTGCTAT-3′) and exon 5 (5′-TCTGAGCCAATGAGGAGACC-3′).

### Statistical Analysis

All values are expressed as mean ± S.D. Statistical significance was determined using a two-sided Student’s t-test and GraphPad Prism software (GraphPad Software, version 4.0; San Diego, CA). Unless stated otherwise, a value of p<0.05 was considered significant.

## Results

### Cellular Levels of Syntaxin 16 Increase with Confluency

**Figure 1:**
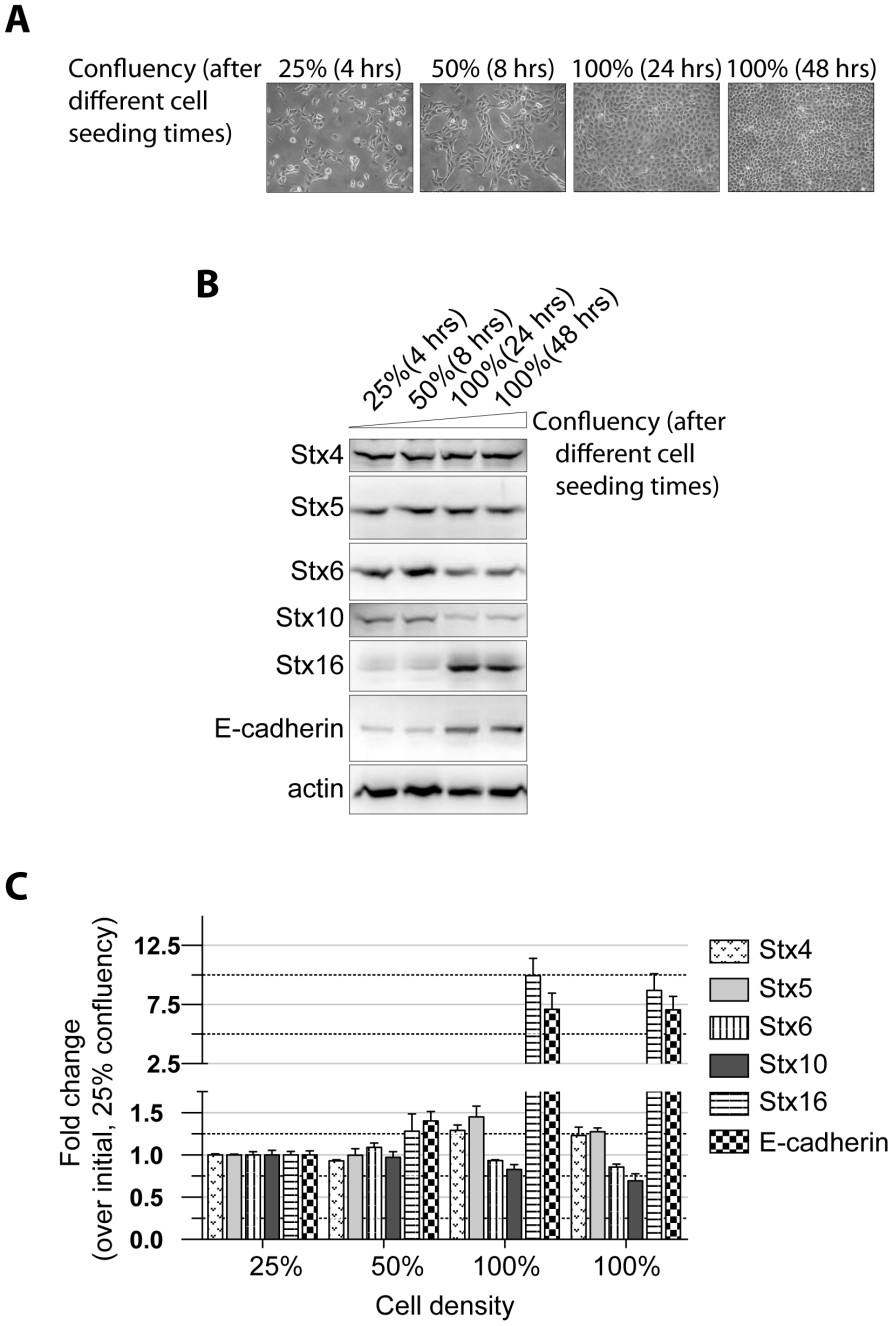
Expression of Stx proteins in MDCK cells over the development and maintenance of a confluent monolayer. (**A–C**) MDCK cells (3.5×10^5^ cells/35 mm dish) were seeded and analyzed after achieving varying degree of confluency. (**A**) Confluency of MDCK monolayers at indicated times after initial cell seeding. Representative images are shown. (**B**) Western blotting for Stx protein and E-cadherin expression. Total cell lysates were prepared from cultures at varying degrees of confluency. (**C**) Quantitation of band densities in ***B***. Values represent relative protein levels at different cell densities, after normalization to the 25%-confluency value, set arbitrarily at 100%. The percentage represents the mean (±SD) for n = 3.

Stx16 is involved in retrograde membrane transport from endosomes to the Golgi complex. We first sought to determine the intracellular levels of syntaxin proteins, both while cell-cell contact is established and once cell-cell contact inhibits cell growth within the epithelial monolayer (Figure 1A). MDCK cells seeded at 3.5×10^5^ density were allowed to grow for 4, 8, 24, or 48 hrs. The first three time points correspond to 25%, 50%, and 100% confluency, respectively, and the last to 24 hrs after attaining 100% confluency. We found that relative to the levels of Stx16 in sparse cultures (25–50% confluency), those in confluent cultures were ≥4-fold high, and that this increase coincided with a higher degree of cell-cell contact (Figure 1B,C). The levels of other stx proteins localized at the endosomes and Golgi complex, such as Stx5, Stx10, Stx6, as well as that of the PM-localized Stx4, remained similar in sparse vs confluent cultures. As expected, the increase in cell-cell contact was also associated with a rise in E-cadherin levels. Thus, increased expression of Stx16 correlated positively with E-cadherin expression.

### Syntaxin 16 Regulates Transepithelial Resistance (TER)

**Figure 2:**
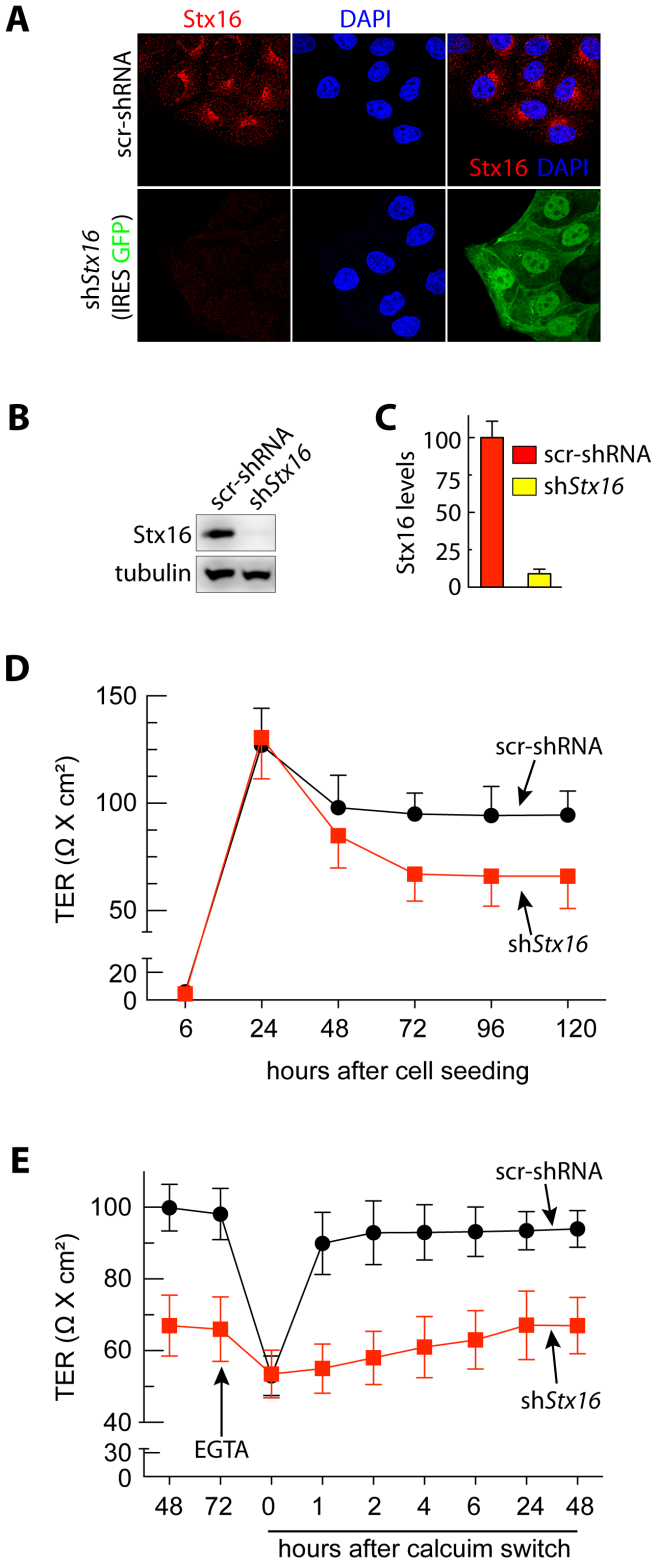
Stx16-depleted MDCK monolayer show decreased transepithelial resistance (TER). (**A**) Immunofluorescence staining for Stx16 expression in src-shRNA-MDCK and sh*Stx16*-MDCK cells. (**B**) Western blotting of Stx16 expression in src-shRNA-MDCK and sh*Stx16*-MDCK cells. (**C**) Quantitation of band densities from ***B***. Values represent relative protein levels after normalization to an arbitrary value of 100% for src-shRNA expressing cells. The percentage represents the mean (±SD) for n = 3. (**D**) Transepithelial resistance (TER) measured in monolayers generated from src-shRNA-MDCK and shStx16-MDCK cells at indicated time intervals. (**E**) Recovery of TER in MDCK monolayers following a Ca^2+^-switch. Stx16 depletion caused a delayed recovery of TER. Graph represents the averages from 3 independent experiments (plus 3 replicates per experiment) and error bars show SEM.

Stx16 is a vesicle fusion protein and involved in the intracellular transport of cargo between endosomes and the Golgi complex [31]. Since the intracellular levels of Stx16 and E-cadherin were positively correlated in sparse and confluent MDCK cells, we investigated the possibility that Stx16 is involved in the trafficking of E-cadherin to maintain stable cell-cell junctions. For this experiment, we transduced MDCK cells with lentiviruses encoding small hairpin RNAs specific for canine *stx16* (sh*stx16*) (Figure 2A). Cells stably expressing sh*stx16* and GFP-reporter were selected using puromycin. Controls stably expressed a scrambled shRNA (scr-shRNA). The extent of Stx16 depletion was determined by immunofluorescence labeling and immunoblotting of endogenous proteins; Stx16 expression at the Golgi was prominent in scr-shRNA-expressing MDCK cells, but significantly reduced in sh*stx16*-MDCK cells (Figure 2A,B) (efficiency of knockdown was ∼95%, Figure 2C).

Since the intracellular levels of Stx16 were elevated when epithelial cells were in contact with neighboring cells we investigated if Stx16 is required for the functional integrity of cell-cell junctions, using TER as a direct indicator. MDCK cells were plated and maintained at confluent density on Transwell filters, forming electrically tight, polarized monolayers. The barrier function of the TJ can be evaluated by measuring the TER of cell monolayers. In the control cell line, the TER values measured began to increase progressively at 6 hrs (Figure 2D). They peaked at 24 hrs and then returned to baseline (Figure 2D). In the sh*stx16*-MDCK cells, the TER also increased along this timeline and returned to baseline (Figure 2D). However, in these cells the baseline TER was consistently maintained at about 30% lower than that in controls (Figure 2D). We then investigated the establishment of cell-cell junctions using a calcium switch assay. E-cadherin-mediated cell-cell contacts were disrupted by treatment with EGTA, a Ca^2+^ chelator, and TJs were then allowed to form by raising extracellular Ca^2+^ levels (calcium switch) [32]. Formation of functional TJs was measured by TER following calcium switch, and found that sh*stx16*-MDCK cells showed a slower rate of recovery of TER in comparison to control cells (Figure 2E). This shows that depletion of Stx16 delays the establishment of cell-cell junctions and impairs epithelial barrier function.

### Syntaxin 16 Regulates Steady-state E-cadherin Accumulation Following the Initiation of Cell-cell Contact

**Figure 3:**
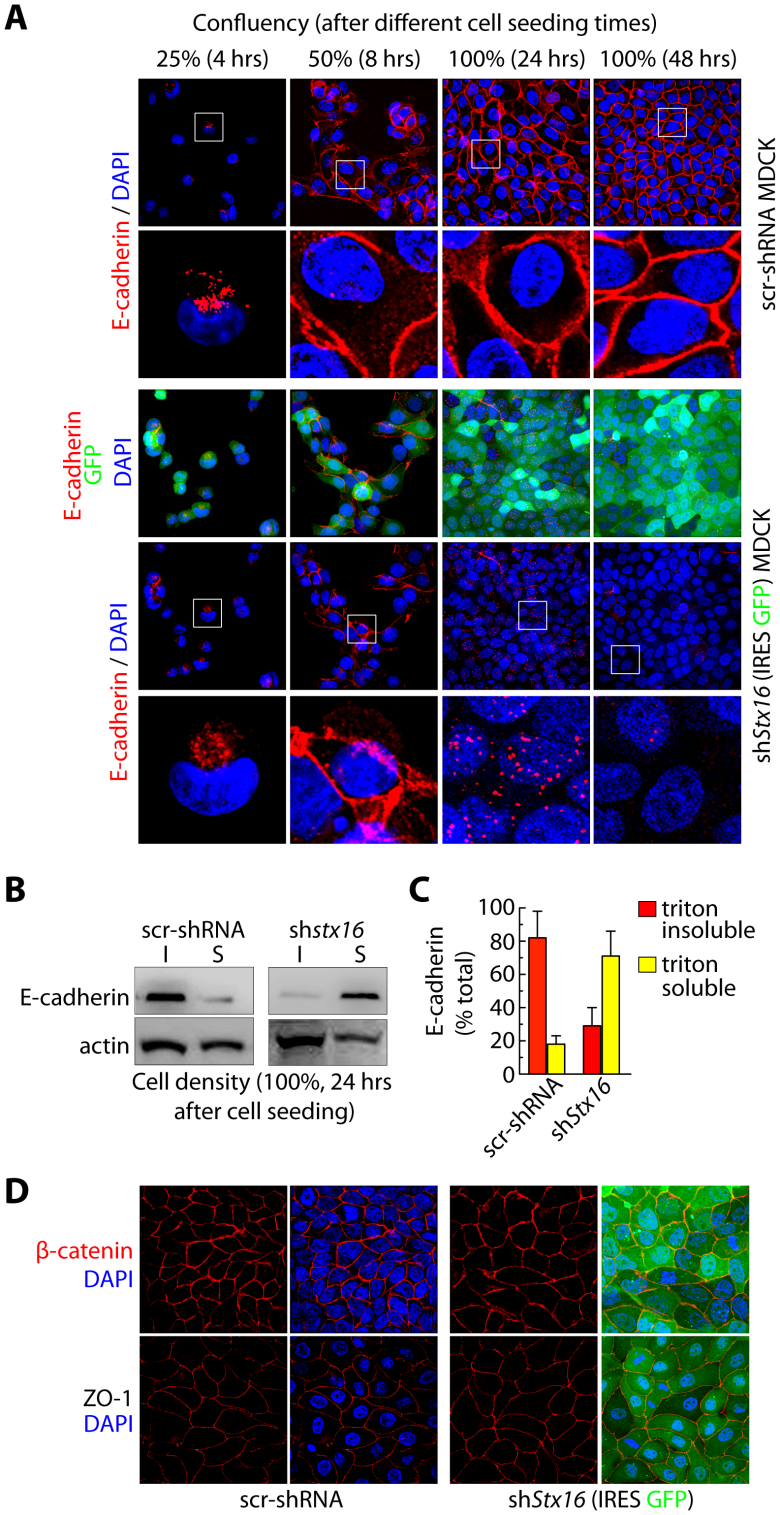
Stx16 depleted MDCK cells show decreased E-cadherin at cell-cell junctions. (**A**) Immunofluorescence-based analysis of E-cadherin localization at MDCK cell-cell junctions during the development and maintenance of a monolayer. (**B**) Western blotting for E-cadherin following preparation of Triton-soluble (*S*) and -insoluble (*I*) fractions from scr-shRNA and shStx16 MDCK confluent monolayer 24 hrs after cell seeding. (**C**) Densitometric quantitation of E-cadherin bands as in ***B***. Values represent the relative protein levels after normalization to an arbitrary value of 100% for src-shRNA expressing cells. The percentage represents the mean (±SD) for n = 3. (**D**) Immunofluorescence-based analysis of -catenin localization in scr-shRNA and shStx16 MDCK at various times post cell seeding.

Since our TER measurements following the calcium switch suggested that junctional integrity is compromised in stx16-depleted cells, we assessed the localization of AJ and TJ proteins before and after initiation of cell-cell contact. As expected, in control cells the level of E-cadherin increased at cell-cell junctions as the cells attained confluency (Figure 3A). In Stx16-depleted cells, however, E-cadherin levels at the these sites were significantly decreased 24 hrs after cell seeding (i.e., at 100% confluency), with a concomitant increase in cytoplasmic vesicular staining (Figure 3A, see enlarged inset). At later times (48 hrs post seeding) overall total E-cadherin staining was also significantly reduced in sh*stx16*-expressing MDCK monolayers relative to controls. These data demonstrate that E-cadherin initially localizes to sites of cell-cell contact in Stx16-depleted MDCK monolayers, but that this localization is not maintained. The localization of ZO-1 (TJ associated protein) at cell-cell junction was not altered in Stx16-depleted MDCK monolayers (Figure 3D). Furthermore, the association of PM-localized cadherin with the cortical actin cytoskeleton is indispensable for strong cell-cell interactions. To analyze the amount of E-cadherin associated with the cytoskeleton, the distribution was determined in Triton X-100-fractionated cell lysates. Triton X-100 fractionation separates cytoskeletal and cytoskeleton-associated proteins, which are present in the Triton-insoluble fraction, from soluble and cytoplasmic proteins, enclosed in the Triton-soluble fraction [33]. After 24 hrs of cell seeding E-cadherin localizes at cell-cell junctions (Figure 3A) where we expect it to associate with cortical actin cytoskeleton. Triton X-100 fractionated cell lysates demonstrate that ≥80% of the total E-cadherin is in insoluble fraction (Figure 3B,C). On the contrary, in Stx16-depleted MDCK cells 70% of the total E-cadherin was in Triton X-100 soluble fraction. The decrease in the amount of E-cadherin in the Triton-insoluble fraction of the sh*Stx16*-expressing cells suggests significantly less cytoskeleton associated E-cadherin. We expected to also find that loss of E-cadherin localization at cell-cell junction leads to a decrease in levels of the E-cadherin-binding protein β-catenin at the AJs. However, levels of β-catenin at the AJs were similar in stx16-depleted and control MDCK monolayers even once the cells attained confluency (Figure 3D). Our results suggest that depletion of Stx16 accumulates E-cadherin intracellularly, however, cell-cell contacts are still maintained.

### Depletion of Syntaxin 16 Reduces E-cadherin Recycling to the Plasma Membrane

**Figure 4:**
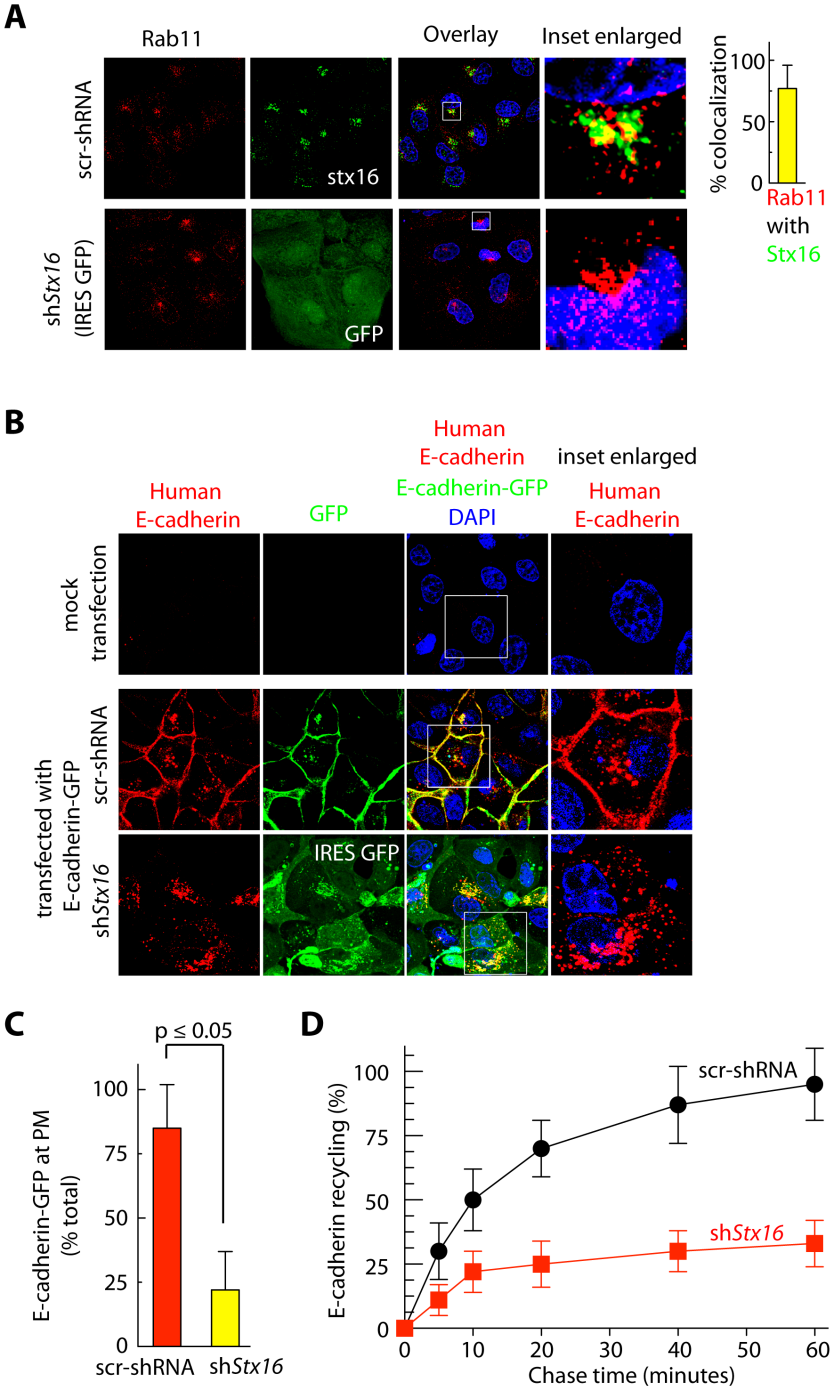
Stx16 colocalizes with Rab11 and E-cadherin in MDCK cells. (**A**) Immunofluorescence based assessment of Rab11 localization in control (src-shRNA) and shStx16-expressing MDCK cells. Graph show quantification of overlap in Stx16 and Rab11 fluorescence signals, from images as in ***A***. Value represents mean ± S.D. (n = 50 cells for each condition, from 5 separate experiments; p≤0.05). Intracellular localization of Rab11 is not altered in Stx16-depleted MDCK cells. (**B**) Immunofluorescence-based analysis of localization of E-cadherin-GFP in cells stably expressing Src-shRNA or sh*Stx16*. (**C**) Quantitation of relative levels of E-cadherin-GFP at the PM (staining with human E-cadherin antibody) along the cell edges vs. total throughout the cell, quantified using the Metamorph software. Values represent relative change in the levels of E-cadherin-GFP at the cell periphery (calculated as % of total cell-associated protein). Results are expressed as mean ± SEM (n = 70 cells for each condition, from three separate experiments and p≤0.05). (**D**) MDCK cells stably expressing scr-shRNA or sh*Stx16* were surface biotinylated. Samples were then incubated at 16°C for 30 min, to allow biotinylated E-cadherin to accumulate in endosomes. Biotin remaining at the surface was removed by treatment with MesNa and quenching of MesNa with iodoacetamide (0-min time point); samples were further incubated at 37°C for the indicated periods and, at each time point shown, subjected to a second MesNa treatment and then assessed for recycling of internalized E-cadherin. After each time point, the cells were lysed and the amount of biotinylated E-cadherin was determined by capture ELISA, using an Ab against E-cadherin. The fraction of internalized E-cadherin recycled back to the PM is expressed as a percentage of surface-labeled protein originally internalized (from the 0-min chase time point). Values are mean ± S.D. from 3 independent experiments; p≤0.005.

Stx16 is known to regulate retrograde cargo transport from early endosomes and recycling endosomes to the Golgi complex [34], [35], [36]. Our results further show that it extensively colocalizes with Rab11 in the perinuclear region (Figure 4A). Given that Rab11 and recycling endosomes are required for recycling and turnover of E-cadherin at the PM [1], [18], and Stx16 has been shown to regulate recycling of both Glut-4 and CFTR [37], [38], we next investigated if Stx16 loss results in a decrease in recycling of E-cadherin to the PM. For this we overexpressed E-cadherin-GFP in MDCK cells, and determined if Stx16 depletion altered E-cadherin-GFP levels at the PM. Since sh*Stx16*-expressing MDCK cells also express GFP reporter we used an antibody that specifically recognizes human E-cadherin (but not the canine E-cadherin) to localize ectopically expressed human E-cadherin-GFP (Figure 4B). Control cells expressing E-cadherin-GFP show typical pattern of endogenous E-cadherin staining with cobblestone boundaries and some intracellular staining (Figure 4B). In contrast, Stx16-depleted cells showed mislocalization of human E-cadherin-GFP with ≥3-fold reduced levels at the PM relative to controls (Figure 4C). Instead, majority of E-cadherin-GFP was concentrated intracellularly (Figure 4B). These results suggest that Stx16 is required for the delivery of E-cadherin to the PM.

To further determine role for Stx16 in the endocytic recycling of endogenous E-cadherin, cells were exposed to biotin and then subjected to a 16°C temperature block, during which surface-biotinylated E-cadherin were internalized and accumulate in early endosomes. Residual surface biotin was removed by treatment with MesNa, and then a sample was taken to establish how much of the initial PM-localized pool of E-cadherin had been internalized. Cells were then subjected to a 37°C chase to allow subsequent trafficking from endosomes. After each chase time point, the samples were again treated with MesNa to remove any biotin from previously internalized E-cadherin that had recycled back to the cell surface, and the level of biotinylated E-cadherin remaining within the cell was quantified by capture ELISA. The percentage of E-cadherin that had recycled back to the PM was determined by comparing the levels of biotinylated E-cadherin remaining after the chase to the amount in the pool internalized originally.

In control (scr-shRNA) cells, almost 50% of the internalized E-cadherin was recycled back to the PM after 10 min of chase, and this number increased to nearly 95% by 60 min of chase (Figure 4D). In contrast, in the context of Stx16 depletion, recycling of E-cadherin during the initial 10 min and later 60 min of the chase period was 22% and 33%, respectively (Figure 4D). Our results suggest that Stx16 plays a role in sorting E-cadherin from endosomes for recycling to the PM.

### Syntaxin 16 is Required for Formation of a Single Lumen During Cystogenesis

Single MDCK cells suspended in Matrigel proliferate to form fluid-filled cysts consisting of a monolayer of polarized cells enclosing a central lumen. The formation of these structures reveals many features of the cells undergoing morphogenesis into polarized epithelia [39]. Confocal immunofluorescence microscopy of cysts confirmed that unlike the MDCK cells in sparse cultures, those forming cysts exhibited high levels Stx16 expression (data not shown).

**Figure 5:**
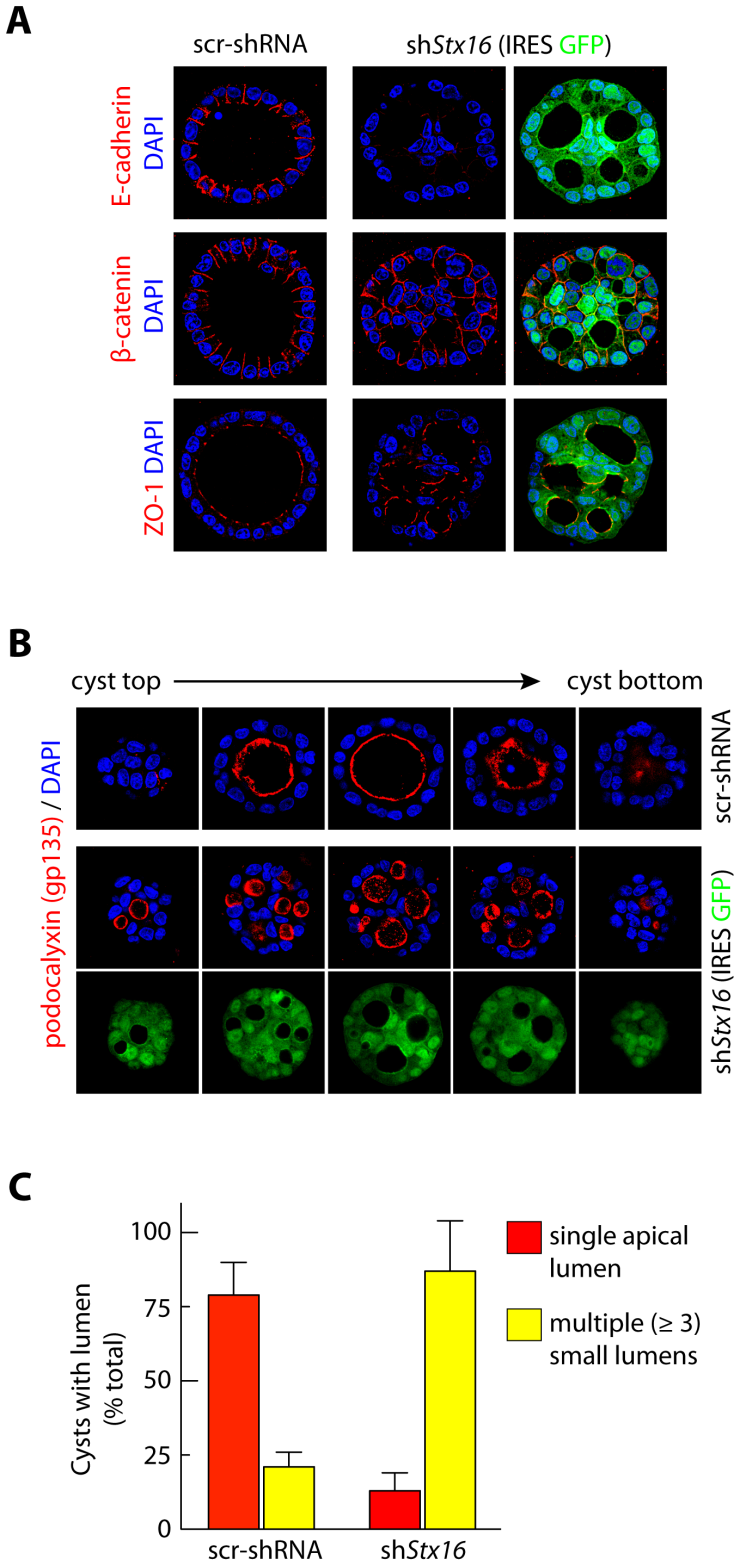
Stx16 depletion leads to decrease in E-cadherin levels and formation of multiple lumens in epithelial cysts. (**A, B**) Immunoflourescence-based analysis of morphology and localization of basolateral and apical proteins in cysts formed from MDCK cells stably expressing scr-shRNA or sh*Stx16* 4d after plating in Matrigel. Cysts were fixed and immunostained for E-cadherin, β-catenin, ZO-1, and podocalyxin (gp135), and DNA was visualized using DAPI. Scale bar, 5 µm. Representative cysts; confocal sections through the top to bottom of a cyst are shown. (**C**) Quantification of cysts with single or multiple lumens as in ***A***, ***B***. Values are mean ± SD from three independent experiments; n >100 cysts/experiment. p<0.01.

Cell polarization and cell-cell adhesion are important events that regulate the formation of a cyst with a single apical lumen [39]. Thus, we next analyzed if cell polarity was affected in Stx16-depleted cysts. Stx16 knockdown did not alter the basolateral localization of ZO-1 and β-catenin (Figure 5A). However, the level of E-cadherin in Stx16-depleted cysts was reduced relative to that in controls (Figure 5A, top panel). Cells in MDCK cysts normally orient their apical surface toward the central lumen [39], and immunofluorescence analysis of control MDCK cysts revealed that >75% had a single lumen faced by the apical side of the cell, i.e., as marked by gp135 (podocalyxin) and phalloidin (cortical-actin; data not shown). However, in the case of cysts formed from Stx16-depleted MDCK cells, >75% featured multiple lumens (>4) (Figure 5B,C). Interestingly, the localization of gp135 and the TJ protein ZO-1 were not altered in the individual multi-lumen cysts (Figure 5A,B). These data suggest that Stx16 is not required for maintaining apical-basal polarity and TJs during MDCK morphogenesis, but rather for correct positioning of the apical surface with respect to the growing cyst structure.

### Syntaxin 16 is Required for Proper Spindle Orientation in the Developing Cyst

**Figure 6:**
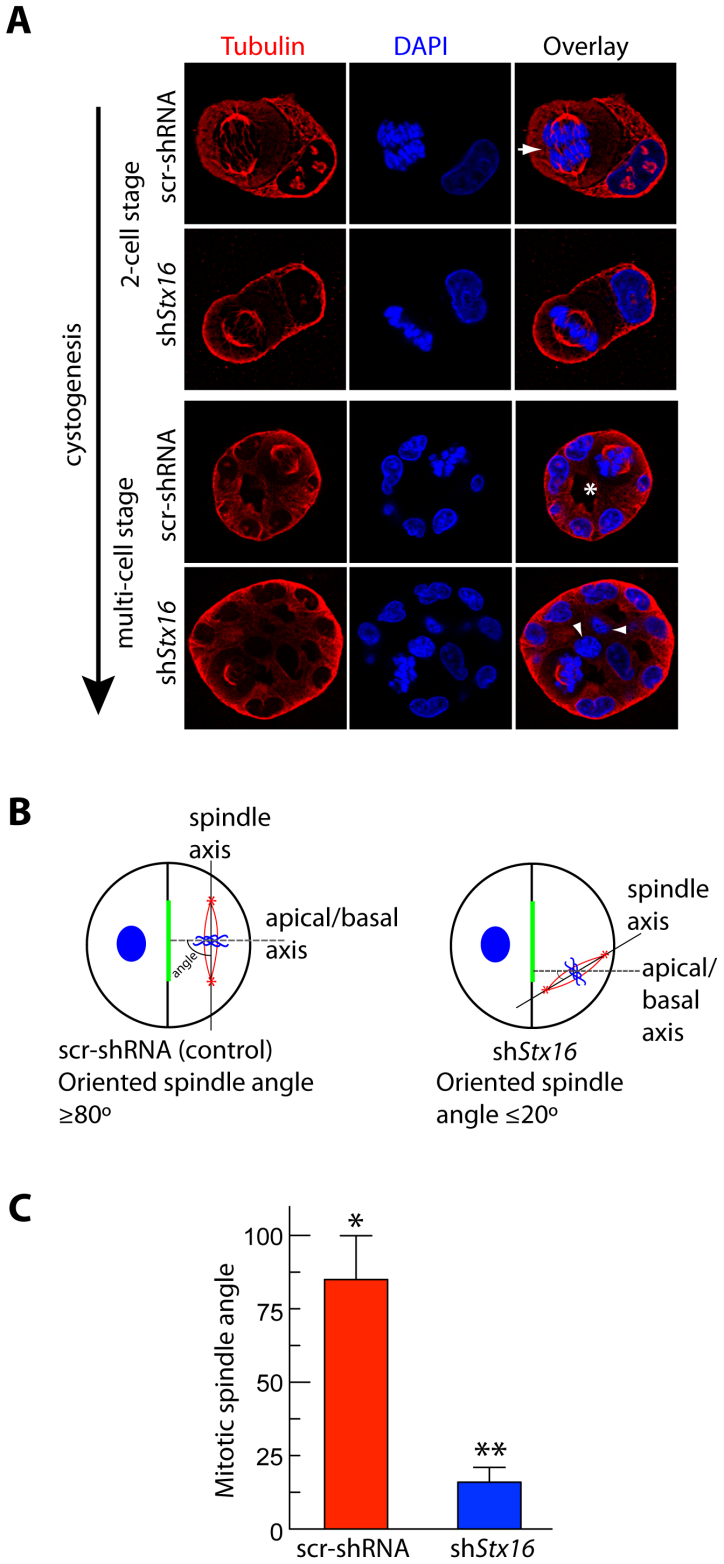
Stx16 depletion leads to spindle misorientation and cystogenesis defects. (**A**) Confocal images of metaphase cells in the middle region of the cysts grown for 1–4 d, following fixation and staining with anti-α-tubulin antibody (red) and DAPI (blue). Note that in control cysts: orientation of the spindle with respect to the apical-basal axis is perpendicular (arrow);cell division occurs in the plane of the monolayer (2-cell stage); abscission is asymmetrical; and deposition of nascent apical surface occurs around a single central lumen (asterisk, multi-cell stage). (**B**) Schematic illustration of spindle-angle measurement. A line was drawn to connect the two spindle poles (thick black line). Another was drawn from the centroid of the apical domain to the midpoint of the spindle axis (thin black lines), and the acute angle (red) between the two lines was assessed. (**C**) Quantification of metaphase spindle angles in MDCK cells stably expressing scr-shRNA or sh*Stx16*. Data show means ± SEM, n  = 70 cysts from three independent experiments. Statistical significance was evaluated using a Mann-Whitney test. *, *p*≤0.002; **, *p*≤0.001.

In some epithelia, spindle orientation plays an important role in the formation of a single apical lumen [40]. To assess whether the formation of multi-lumen cysts in Stx16-depleted MDCK cells is associated with disruption of spindle orientation, we examined cysts grown from control and sh*Stx16*-MDCK cells in three dimensional (3D) culture (in Matrigel) for the orientation of the mitotic spindles in the cells. Comparison of spindle orientation during both the early-aggregate (2-cell) and open lumen stages of cystogenesis revealed that stx16 depletion had a dramatic effect (Figure 6). Whereas in control cysts the majority of spindles were oriented perpendicular to the centroid (mean angle ≥80°), in cysts formed from stx16-depleted cells, spindle orientation was random (mean angle ≤20°), with some spindles oriented so as to produce a daughter cell in the middle of the structure (Figure 6A,B,C).

### Syntaxin 16 is Required for Zebrafish Pronephric-duct Morphogenesis

**Figure 7:**
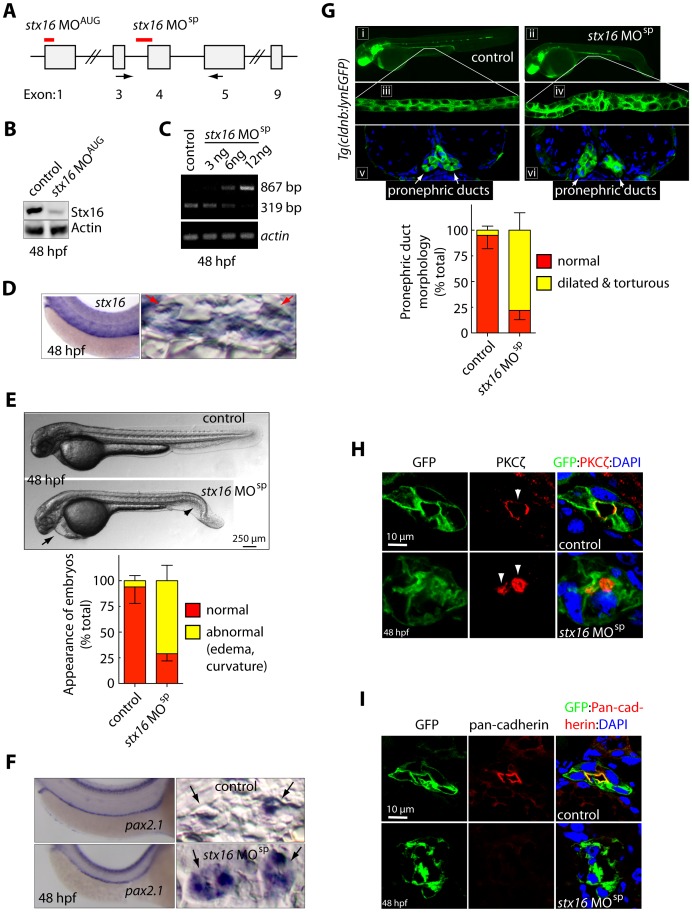
Stx16 depletion causes abnormal pronephric-duct formation in zebrafish. (**A**) Diagram of the exon and intron organization of zebrafish *stx16*. Thick red lines indicate the binding regions of two antisense morpholinos (MOs), one of which targets the start codon (AUG) to block translation of *stx16*, and the other the mRNA splice acceptor site of exon 4 (splicing) to block splicing of the *stx16* mRNA. The two arrows indicate the locations of primers used in RT-PCR analysis in control and *stx16* morphants. (**B**) Western blot analysis showing the expression of Stx16 in control and *stx16*-MO^AUG^-injected embryos at 48 hpf; expression of alpha-actin served as a loading control. (**C**) RT-PCR showing 319 bp and 867 bp amplicons, which result, respectively, from amplification from exon 4 and exon 4 plus unspliced intron 3 in the control uninjected and *stx16*-MO^sp^-injected embryos. (**D**) Expression of *stx16* at 48 hrs, as detected by ISH. Left-hand panel: lateral view with anterior to the left. Right-hand panel: cross section of the region shown to left. Arrow: pronephric duct. (**E**) Normarski images (lateral view) of control and *stx16* MO^sp^-injected embryos at 48 hpf. Bar graph at right shows the percentage of embryos showing abnormalities (edema and curvature). Arrow: pericardial edema; arrowhead: body curvature. (**F**) *pax2.1* expression at 48 hpf, as detected by ISH in the indicated embryos. Left-hand panels: Lateral view of the trunk region of indicated embryos, with anterior to the left. Right-hand panels: Transverse sections of embryos of the left-hand panel. Arrows: pronephric duct. (**G**) Top panel (i, ii): Representative epifluorescence images of 48-hpf *Tg(cldnb:lynEGFP)* uninjected control and *stx16*-MO^sp^-injected embryos. Lateral view, anterior to the right, pronephric ducts. Middle panel (iii, iv): Confocal Z-stack images of pronephric ducts of *Tg(cldnb:lynEGFP)* taken at the regions shown in top panel. Bottom panel (v, vi): transverse sections of *Tg(cldnb:lynEGFP)* embryos showing the pronephric ducts (arrows). Nucleoli are stained with DAPI. Graph showing the percentages of embryos exhibiting pronephric-duct dilation. (**H**, **I**) Confocal (Z-stack) images of transverse sections of *Tg(cldnb:lynEGFP)* embryos at 48 hpf, showing the expression of PKCζ and pan-cadherin on one side of pronephric duct. Arrowheads: lumens.

To investigate the role of Stx16 *in vivo*, we explored its function in zebrafish kidney morphogenesis. A homolog of human *Stx16* in zebrafish shares 81% homology at the amino-acid level, and 73% homology at the nucleic-acid level, as referenced in HomoloGene in the NCBI database. Whole-mount *in situ* hybridization revealed that, consistent with the expression pattern of its human homolog [36], [41], [42], zebrafish *stx16* is ubiquitously expressed in the embryo, including in the pronephric duct, which becomes the kidney in zebrafish (Figure 7D) [43]. Thus, Stx16 may play a role in kidney development in zebrafish. We tested this possibility by using two different MOs to inhibit Stx16 expression: MO^AUG^ was designed to block translation initiation of *stx16*, and MO^sp^ to block mRNA splicing by targeting the splice acceptor site of exon 4 (intron 3-exon 4), which leads to insertion of intron 3 and introduces a stop codon. The truncated product lacks the C-terminal transmembrane region as well as a central SNARE domain which is critical for its function. We found the expression level of the Stx16 protein was significantly reduced in embryos injected with *stx16*-MO^AUG^ at 48 hpf as compared to that in uninjected control embryos (Figure 7B). RT-PCR showed that embryos injected with *stx16*-MO^sp^ had an abnormal amplicon of 867 bp, which corresponds to insertion of intron 3, and reduced levels of the normally spliced 319 bp amplicon between exon 3 and exon 5 (the extent of this effect was dependent on the amount of MO^sp^ injected; Figure 7C). These results indicate that both MOs effectively inhibit Stx16 expression in zebrafish.

To assess the function of Stx16 in zebrafish kidney morphogenesis, we used *Tg(cldnb:lynGFP)* transgenic fish, which express membrane-bound GFP placed under the control of the Claudin B promoter [24]. Claudin B is a member of the tetraspanin family of tight junction proteins that is transcribed at high levels in organs derived from sensory placodes, including the pronephric ducts [25]. At 48 hpf, embryos injected with either *stx16*-MO^AUG^ or *stx16*-MO^sp^ (morphants) displayed abnormal body curvature and pericardial edema (71% of 50 morphants) (Figure 7E, Figure S1). Interestingly, in the morphants the pronephric duct (as visualized by mGFP expression) was dilated and torturous (78% of 50 morphants) (Figure 7G). The pericardial edema associated with this phenotype progressed as the embryos developed, and most did not survive past 4–5 dpf. Both the severity and penetrance of the observed phenotypes increased with the dose of MO (data not shown). Furthermore, *pax2.1*, an early-stage marker of pronephros development [44], [45] was expressed in the enlarged pronephric ducts of *stx16*-morphants (Figure 7F), suggesting that Stx16 is required for the morphogenesis, but not differentiation, of this structure.

To further examine how *stx16* affects development of the pronephric duct, we performed confocal imaging in fixed *Tg(cldnb:lynGFP)* embryos. Consistent with findings from previous studies, the control embryos had a clear pronephric-duct structure, with single lumen at 48 hpf. In contrast, *stx16*-morphants had a significantly enlarged and torturous pronephric duct with abnormal luminal structures, including multiple lumens (Figure 7H). As cells of the pronephric duct display characteristic apical-basal polarity with a central lumen, we next asked if this organization is disrupted in *stx16* morphants. We performed an immunofluorescence assay on transverse cryosections through the pronephric duct, localizing markers of the apical and lateral membranes, e.g., atypical protein kinase C (PKCζ) and pan-cadherin, respectively, to assess lumen morphology and cell-junction integrity. Strikingly, in *stx16*-morphants, staining of cross sections of the pronephric duct for the atypical protein kinase (PKCζ) revealed two lumens, whereas the corresponding control sections showed a relatively large single lumen marked by intense staining with the PKCζ antibody at the apical membrane (facing the duct; Figure 7H, Figure S1). Furthermore, cadherin expression in pronephric-duct sections from *stx16*-morphants showed reduced staining relative to controls (Figure 7I). The *in vivo* effect of *stx16* depletion on duct-lumen morphology and cadherin expression was consistent with our findings from the *in vitro* 3D cyst cultures (Figure 5), further supporting our hypothesis that Stx16 is required for maintaining a single-lumen structure and cadherin expression at cell junctions during epithelial morphogenesis.

## Discussion

Membrane trafficking is critical for the formation of an apical lumen from an epithelial sheet [46], [47], [48], [49]. Retrograde membrane transport from endosomes to the Golgi complex has been shown to require Stx vesicle-fusion proteins [50], [51], [52]. Among the family members known to regulate retrograde cargo transport between endosomes and the Golgi in non-polarized cells are Stx5, Stx6, Stx10, and Stx16. Stx16 in particular has been shown to regulate transport from endosomes to the Golgi complex and recycling endosomes [35], [36], [37], [38]. The role of endosome and Golgi localized stx proteins in E-cadherin trafficking and epithelial morphogenesis is less understood. In this study using MDCK cells, we show that intracellular Stx16 levels increase in monolayers and in 3D cysts, and that this protein is required for maintaining E-cadherin at epithelial cell-cell junctions. We show that Stx16 is required for proper spindle orientation and formation of a single apical lumen. Our findings reveal that Stx16 depletion causes E-cadherin degradation in lysosomes, altered spindle orientation during the proliferation involved in cystogenesis in Matrigel, and the formation of multiple lumens within the cyst. In addition, they show that zebrafish require stx16 to form normal pronephric ducts.

During trafficking to and from cell-cell junctions, E-cadherin-catenin complexes are dynamically assembled and disassembled [53]. In several epithelial cell lines, this redistribution of E-cadherin is carried out largely by endocytosis [53], [54]. Moreover, different routes of E-cadherin endocytosis make it possible to achieve trafficking either concomitant with or in the absence of an influence on adhesion [54]. E-cadherin-based cell-cell adhesion is regulated by delivery and turnover of E-cadherin at the PM [53], [54], and one of the mechanisms that regulates E-cadherin turnover at the PM involves the recycling endosomes [55], [56]. In polarized epithelial cells, cargo transport for recycling from these structures is regulated by Rab 11 [18], [47]. Our results show that in confluent cultures of control cells, Stx16 extensively colocalize with Rab11, and that in Stx16-depleted cells E-cadherin is unable to recycle to the PM. Stx16 has been shown to regulate Glut-4 recycling and CFTR recycling in adipocytes and epithelial cell types, respectively [37], [38]. These findings suggest that Stx16 regulate E-cadherin recycling by influencing the delivery of E-cadherin-containing transport vesicles to the recycling endosomes.

Stable junctions provide cues for epithelial lumen formation, a process that is complex and relies on cellular machineries that have to function in parallel [46]. In the context of Stx16 loss, intact cell junctions were observed and TER did not drop altogether, in spite of the fact that cell junctions had significantly reduced E-cadherin levels. This is supported by our data that Stx16 depletion did not alter the cellular distribution of the TJ-associated protein ZO-1. Alternatively, loss of functional E-cadherin due to Stx16 depletion might have been compensated by the presence of cadherin-6, which is also expressed at high levels in MDCK cells [57]. Previous studies have shown that E-cadherin depletion disrupts the establishment, but not maintenance, of cell junctions [58]. Our analysis of E-cadherin expression during cystogenesis by stx16-depleted cells suggest Stx16 is not require for the recruitment of E-cadherin to junctions, but rather for its maintenance at this site, probably through its involvement in E-cadherin recycling. Although Stx16 depletion does not seem to alter the gross morphology of epithelial cells in confluent monolayers, it severely affected lumen formation during cystogenesis. Our data suggest that Stx16 is an essential player in maintaining a single lumen in epithelial cysts, and that it probably does so by maintaining E-cadherin at the AJs. These conclusions are also consistent with previous studies correlating E-cadherin loss with the formation of multiple lumens [57]. The fact that β-catenin localization was not altered by Stx16 depletion further suggests that in the absence of E-cadherin other cadherin family members (such as cadherin-6) may compensate for loss of E-cadherin and facilitate the recruitment of cadherin-associated proteins to the cell junctions [58], [59], [60]. Although lack of Stx16 results in the formation of multiple lumens, apical polarity with respect to the individual lumen in a cyst was maintained, suggesting that Stx16 does not alter the trafficking of apical markers in polarized cells. In addition, our present findings establish a novel mechanism whereby Stx16 may affect lumen formation during cystogenesis, i.e., by acting as a regulator of E-cadherin trafficking it provides cues that orient the mitotic spindle during symmetric cell divisions in mammalian epithelia [59]. Alternatively, Stx16 may regulate membrane trafficking during cytokinesis that regulates midbody placement and affects lumen origin [61].

Although tissue culture models have provided insights into the role of Stx16 in lumen formation, considering how this mechanism relates to development of the epithelial lumen, we analyzed the role of Stx16 in morphogenesis of the zebrafish pronephric duct. Critical to this process is the development of epithelia from mesenchyme, which leads to duct formation. Although the pronephric-duct structure in zebrafish is distinct from that in higher vertebrates, both tubular and glomerular differentiation are conserved, as are the cellular architecture and transport functions of the pronephric epithelial cells [62]. In *stx16*-morphants, the morphology of pronephric duct was abnormal, featuring an enlarged diameter associated with the presence of a greater number of cells, as well as a torturous, multi-lumen duct structure. Although previous studies have demonstrated a role for zebrafish cadherins 6 and 17 in maintaining pronephric-duct integrity during embryogenesis [63], [64], ours is the first report showing a role for stx16, and that it may exert its effects by regulating cadherin levels at the cell-cell junctions. Taken together our *in vitro*, 3D culture model, and *in vivo* results demonstrate that Stx16 plays a role in morphogenesis of the epithelial lumen.

## Supporting Information

Figure S1 **Depletion of Stx16 by** ***stx16*** **MO^AUG^ causes abnormal body curvature and pronephric-duct formation in zebrafish.** (**A**) Normarski images (lateral view) of control and *stx16* MO^AUG^-injected embryos at 48 hpf. Bar graph at right shows the percentage of embryos showing abnormalities (edema and curvature). Arrow: pericardial edema; arrowhead: body curvature. (**B**) Confocal images of transverse sections of *Tg(cldnb:lynEGFP)* embryos at 48 hpf, showing the expression of PKCζ on one side of pronephric duct. Arrowheads: lumens.
(TIF)
