# Supplementary figures and images for: Syntaxin 16 Regulates Lumen Formation during Epithelial Morphogenesis
Source: PLoS One. 2013 Apr 23;8(4):e61857. doi: 10.1371/journal.pone.0061857 (PMC3633931; doi:10.1371/journal.pone.0061857)

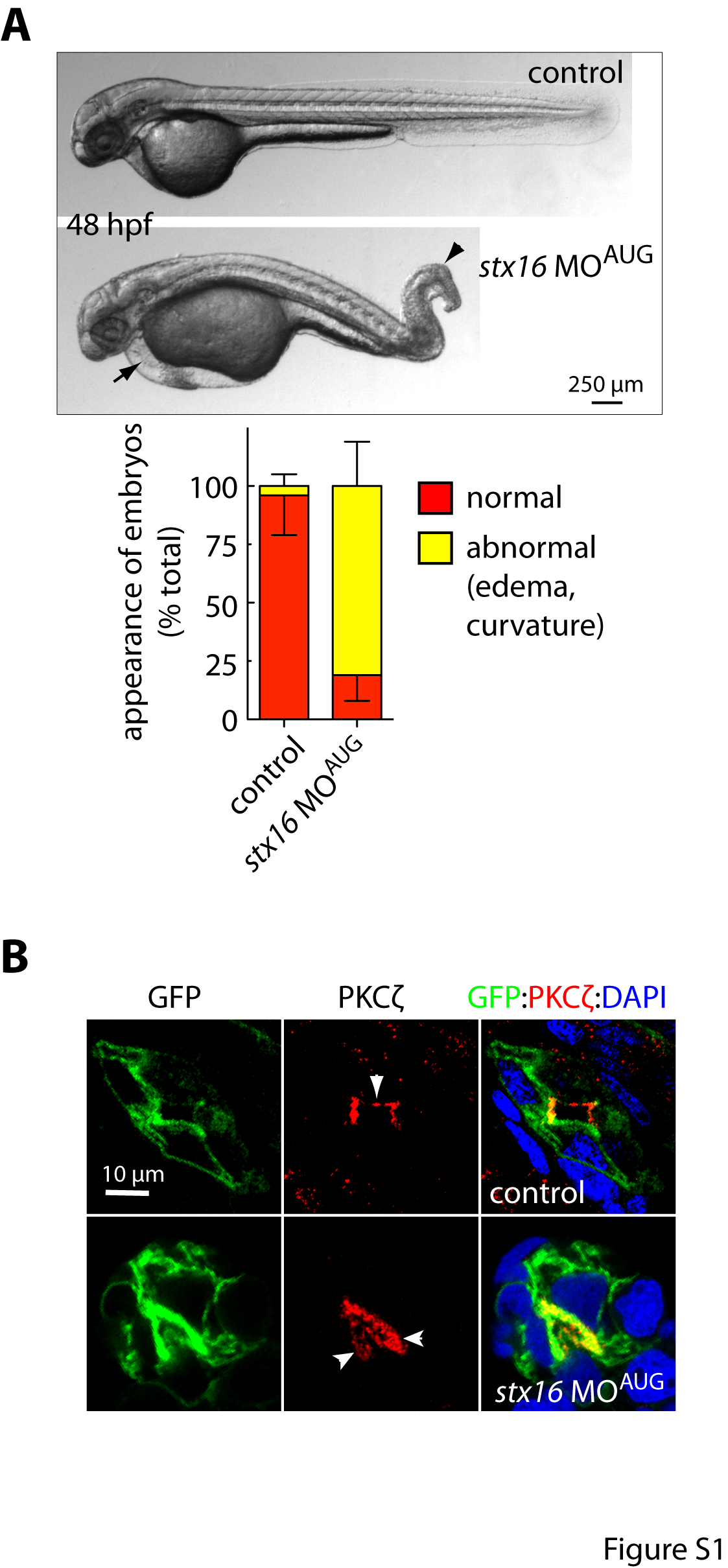

Supplement: Figure S1 — Depletion of Stx16 by stx16 MOAUG causes abnormal body curvature and pronephric-duct formation in zebrafish. (A) Normarski images (lateral view) of control and stx16 MOAUG-injected embryos at 48 hpf. Bar graph at right shows the percentage of embryos showing abnormalities (edema and curvature). Arrow: pericardial edema; arrowhead: body curvature. (B) Confocal images of transverse sections of Tg(cldnb:lynEGFP) embryos at 48 hpf, showing the expression of PKCζ on one side of pronephric duct. Arrowheads: lumens. (TIF) [file pone.0061857.s001.tif]
